# Genome Reduction for Niche Association in *Campylobacter Hepaticus*, A Cause of Spotty Liver Disease in Poultry

**DOI:** 10.3389/fcimb.2017.00354

**Published:** 2017-08-11

**Authors:** Liljana Petrovska, Yue Tang, Melissa J. Jansen van Rensburg, Shaun Cawthraw, Javier Nunez, Samuel K. Sheppard, Richard J. Ellis, Adrian M. Whatmore, Tim R. Crawshaw, Richard M. Irvine

**Affiliations:** ^1^Bacteriology, Animal and Plant Health Agency Weybridge Addlestone, United Kingdom; ^2^Department of Zoology, University of Oxford Oxford, United Kingdom; ^3^NIHR Health Protection Research Unit in Gastrointestinal Infections, University of Oxford Oxford, United Kingdom; ^4^Veterinary Surveillance, Animal and Plant Health Agency Weybridge Addlestone, United Kingdom; ^5^Department of Biology and Biotechnology, The Milner Centre for Evolution, University of Bath Bath, United Kingdom

**Keywords:** *Campylobacter hepaticus*, spotty liver disease, poultry, genome reduction, niche adaptation

## Abstract

The term “spotty liver disease” (SLD) has been used since the late 1990s for a condition seen in the UK and Australia that primarily affects free range laying hens around peak lay, causing acute mortality and a fall in egg production. A novel thermophilic SLD-associated *Campylobacter* was reported in the United Kingdom (UK) in 2015. Subsequently, similar isolates occurring in Australia were formally described as a new species, *Campylobacter hepaticus*. We describe the comparative genomics of 10 *C. hepaticus* isolates recovered from 5 geographically distinct poultry holdings in the UK between 2010 and 2012. Hierarchical gene-by-gene analyses of the study isolates and representatives of 24 known *Campylobacter* species indicated that *C. hepaticus* is most closely related to the major pathogens *Campylobacter jejuni* and *Campylobacter coli*. We observed low levels of within-farm variation, even between isolates collected over almost 3 years. With respect to *C. hepaticus* genome features, we noted that the study isolates had a ~140 Kb reduction in genome size, ~144 fewer genes, and a lower GC content compared to *C. jejuni*. The most notable reduction was in the subsystem containing genes for iron acquisition and metabolism, supported by reduced growth of *C. hepaticus* in an iron depletion assay. Genome reduction is common among many pathogens and in *C. hepaticus* has likely been driven at least in part by specialization following the occupation of a new niche, the chicken liver.

## Introduction

Spotty liver disease (SLD) is an important concern for the poultry egg and meat industries. The disease is sporadic in nature and predominantly affects free-range laying hens, causing a drop in egg production, and up to 10% mortality in some flocks (Grimes and Reece, 2011; Jennings et al., 2011; Crawshaw et al., 2015). SLD is characterized by the appearance of 1–2 mm gray/white foci in the liver, described as multifocal fibrinogranulocytic necrotising hepatitis when examined microscopically (Crawshaw et al., 2015). There are similarities between the epidemiology and pathology of SLD and vibrionic hepatitis, with the terms used interchangeably (Jennings et al., 2011). Vibrionic hepatitis was first described in the United States of America in the 1950s (Tudor, 1954) but appears to have declined since the 1960s (Shane and Stern, 2003). A *Vibrio*-like organism was isolated from cases of vibrionic hepatitis and disease was reproduced (Delaplane et al., 1955; Winterfield and Sevoian, 1957); however, the organism was never fully characterized and questions remain about the nature of this disease and how it relates to the contemporary manifestations of SLD.

Although our understanding of SLD remains limited, recent studies have implicated *Campylobacter* as an etiological agent. The microscopic pathology of SLD was reproduced in specific pathogen-free (SPF) chicks experimentally infected with a novel thermophilic *Campylobacter* isolated from SLD cases in the UK (Crawshaw et al., 2015). Analysis of 16S rRNA gene sequences suggested that the SLD-associated *Campylobacter* isolates represented a novel species within the genus. These organisms grouped with the other thermotolerant *Campylobacter* species, showing most pairwise identity with type strains of *C. jejuni, Campylobacter lari* and *Campylobacter subantarticus*, and *Campyloacter insulaenigrae*, members of the *C. lari* group previously isolated from wild birds and marine mammals respectively (Miller et al., 2014a). In 2016, Van *et al*. formally described a novel thermophilic *Campylobacter* species, *C. hepaticus*, which was isolated from cases of SLD in Australia (Van et al., 2016) using the approach pioneered in the UK. *C. hepaticus* was subsequently confirmed to be the causative agent of SLD, with gross liver lesions typical of clinical cases reproduced in mature layer hens (Van et al., 2017). The results of 16S rRNA gene sequencing, supported by phenotypic and biochemical testing, suggested that the novel SLD-associated *Campylobacter* previously isolated in the UK was also *C. hepaticus*. Like other campylobacters, *C. hepaticus* appears to be fastidious in its growth requirements but is much slower growing than the more commonly isolated thermophilic strains, with some colonies reportedly taking up to 7 days incubation to appear (Crawshaw et al., 2015). This fastidiousness and slow growth may account for the failure of previous attempts to identify the causative agent of SLD. As modified protocols with extended incubation times are not commonly used, the prevalence of *C. hepaticus* is likely underappreciated.

Among the diverse members of the *Campylobacter* genus, *C. jejuni* and *C. coli* are the best studied, largely because together they are the leading bacterial causes of human gastroenteritis worldwide (Kaakoush et al., 2015). Human disease is strongly associated with the consumption of contaminated poultry (Sheppard et al., 2009a,b), and several genetic factors have been implicated in the survival of *Campylobacter* outside the host gut (Pascoe et al., 2015; Yahara et al., 2017) and transmission through the food production chain (Yahara et al., 2017). The prevalence and abundance of *Campylobacter* in the chicken gut and the high levels of carcass contamination at slaughter are thought to contribute to the incidence of human disease (Johnsen et al., 2006; Luber and Bartelt, 2007). Control of *Campylobacter* in chickens is therefore a potential means for reducing human infection. While human infection is usually thought to cause acute symptoms, *Campylobacter* has generally been regarded a commensal of chicken. However, there is evidence that *C. jejuni* does induce humoral and pro-inflammatory responses in chicken (Cawthraw et al., 1994; Smith et al., 2008) and can cause diarrhea (Cogan et al., 2007; Little et al., 2010) and damage to gut mucosa (Stern et al., 1995; Whyte et al., 2001; Line and Bailey, 2006; Wigley, 2015).

Whole-genome sequencing has provided important insights into the genetics and evolution of *Campylobacter* species that occupy different host niches (Miller et al., 2014a; Gilbert et al., 2016; Graaf-van Bloois et al., 2016; van der Graaf-van Bloois et al., 2016). However, little is known about how variation in species and strains relates to sub-structure within the chicken gut niche, and how this correlates with the emergence of diseases such as SLD. Here, we used a comparative genomics approach to investigate SLD-associated *Campylobacter* isolates sampled in the UK. By describing genomic features, with reference to published *Campylobacter* genomes, we identified potential genetic components that contributed to the diversification of the SLD-associated *Campylobacter* from closely related species and features associated with niche specialization.

## Materials and methods

### Bacterial isolates

Ten putative *C. hepaticus* isolates from the strain collection of the UK Animal and Plant Health Agency (APHA) (Addlestone, UK) were included in this study (Table 1). Nine of these isolates were previously described in a report of a novel *Campylobacter* species associated with SLD (Crawshaw et al., 2015). All isolates were cultured from liver samples collected immediately post-mortem from birds showing signs of SLD. Samples were collected from five distinct holdings in the UK between 2010 and 2012 (Table 1). The farms were in different geographic locations with no known epidemiological links between them. All strains were stored at −80°C in 1% (w/v) protease peptone water containing 10% (v/v) glycerol until required.

**Table 1:** Study isolates.

**Date received**	**Farm**	**Strain ID**	**ENA/NCBI accession**	**References**
26/02/2010	FARM 1	S10-0209	ERS1508458	Crawshaw et al., 2015
01/07/2011	FARM 1	S11-010	ERS1508462	Crawshaw et al., 2015
01/07/2011	FARM 1	S11-5013	ERS1508464	Crawshaw et al., 2015
27/11/2012	FARM 1	S12-1018	ERS1508467	Crawshaw et al., 2015
13/09/2011	FARM 2	S11-0036	ERS1508459	This study
13/09/2011	FARM 2	S11-0038	ERS1508463	Crawshaw et al., 2015
26/01/2012	FARM 3	S12-002	ERS1508465	Crawshaw et al., 2015
09/11/2011	FARM 4	S11-0069	ERS1508460	Crawshaw et al., 2015
09/11/2011	FARM 4	S11-0071	ERS1508461	Crawshaw et al., 2015
25/05/2012	FARM 5	S12-0322	ERS1508466	Crawshaw et al., 2015

### Whole-genome sequencing

Isolates were grown from frozen on 10% (v/v) sheep blood agar plates containing Skirrow's antibiotics [vancomycin (10 mg/mL), polymyxin B (2.5 i.u./mL) trimethoprim (5 mg/mL) and actidione (250 mg/mL)] (BASA; Skirrow, 1977) at 42°C in a microaerobic atmosphere (85% (v/v) N_2_, 7.5% (v/v) CO_2_, 7.5% (v/v) O_2_). The bacteria from each plate were harvested into tubes containing 1 mL 0.1 M PBS (pH 7.2) solution. Cells were pelleted by centrifugation and re-suspended in 0.5 mL 0.1 M PBS (pH 7.2) solution. Genomic DNA was extracted using the ArchivePure DNA Cell/Tissue (1 g) kit (5Prime, Gaithersburg, USA). Extracted genomic DNA was fragmented, tagged for multiplexing with the Nextera XT DNA Sample Preparation Kit (Illumina) and sequenced at the APHA on the Illumina MiSeq platform using 150 bp paired-end reads according to the manufacturer's instructions.

### Relationships between *C. hepaticus* isolates and other campylobacter species

The short-read data were assembled using SPAdes (Bankevich et al., 2012) and the resulting draft assemblies were submitted to the Ribosomal Multilocus Sequence Typing (rMLST) database https://pubmlst.org/rmlst/). Relationships between the study isolates and other *Campylobacter* species were characterized at the rMLST (Jolley et al., 2012) and core-genome multilocus sequence typing (cgMLST) levels (Maiden et al., 2013). Following annotation of the ribosomal protein genes (*rps*) using Bacterial Isolate Genome database (BIGSdb) software implemented on the rMLST database (Jolley and Maiden, 2010), the study isolates were compared to the *C. hepaticus* type strain HV10 (Van et al., 2016) and 76 publicly available genomes comprising 24 *Campylobacter* species (Supplementary Table S1). The *rps* genes were concatenated and aligned using MAFFT version 7.037b (Katoh and Standley, 2013) and a Maximum Likelihood tree was generated with MEGA-CC version 7.0 using the general time-reversible model with gamma-distributed rates plus invariant sites with 100 bootstrap replicates. A higher resolution comparison of the study isolates, HV10, and closely related thermophilic *Campylobacter* species, including *C. jejuni, C. coli, C. upsaliensis, C. cuniculorum, C. lari, C. subantarcticus, C. peloridis, C. volucris*, and *C. ornithocola* (Supplementary Table S1), was carried out using the Genome Comparator module implemented in BIGSdb (Jolley and Maiden, 2010). An *ad hoc* cgMLST analysis was carried out by comparing the isolates to the annotated genome of *C. jejuni* NCTC 11168 (GenBank accession AL111168) (Parkhill et al., 2000; Gundogdu et al., 2007), using the default Genome Comparator settings with the core genome cut-off set to 90%. A Maximum Likelihood tree was reconstructed as described for the rMLST analysis, using the MAFFT alignment of concatenated core gene sequences produced by Genome Comparator.

Currently there is no multilocus sequence typing (MLST) scheme for *C. hepaticus*; therefore, we used a read-mapping approach to quantify the variation in the seven gene fragments that comprise the *C. jejuni*/*coli* MLST scheme, namely *aspA, glnA, gltA, glvA pgm, tkt*, and *uncA* (Dingle et al., 2005). For sequence-read alignment and single nucleotide polymorphism (SNP) detection, paired-end Illumina sequence data were mapped to assembled gene fragments from isolate S12-1018, using BWA (Li and Durbin, 2009). For high-resolution genome-wide SNP detection, sequence data were mapped to the draft genome of HV10 (Van et al., 2016). SNPs were identified using Freebayes (https://github.com/ekg/freebayes) and filtered with a minimum mapping quality of 10 and quality ratio cut-off of 0.9. For phylogenetic analyses, a maximum-likelihood phylogenetic tree was constructed from the SNP alignments after Gubbins was run to remove regions of recombination in the pseudofasta files from SNP calling (Croucher et al., 2015). The phylogenetic trees were built using Figtree as previously described (Petrovska et al., 2016).

### Functional analyses

The draft genomes of HV10 and the UK *C. hepaticus* isolates were annotated using the RAST server (Aziz et al., 2008; Overbeek et al., 2014), as were the finished genomes of *C. jejuni* isolates NCTC 11168, M1, PT14, R14, and 4031 (Supplementary Table S1). FIGfam clusters genes based on protein sequence similarity. These genes are then clustered into hierarchical subsystems that display increasing functional breadth (Overbeek et al., 2005). To compare the subsystems generated by RAST, a Student's *t*-test was performed (two tailed distribution for two-sample populations of unequal variance) using a standard spreadsheet function (Microsoft Excel). *P* ≤ 0.05 were regarded as significant. Genome comparisons of the study isolates, HV10, and *C. jejuni* NCTC 11168 were visualized using the BLAST Ring Image Generator (BRIG; Alikhan et al., 2011).

Further analyses were carried out to identify genes involved in antimicrobial resistance, pathogenicity, and iron uptake and metabolism. The SRST2 pipeline (Inouye et al., 2014) was used to search for determinants in the ARG-ANNOT antimicrobial resistance database (Gupta et al., 2014). Putative pathogenicity genes were predicted with the PathogenFinder web-server (Cosentino et al., 2013) using the “All” model. Blastn was used to search for iron uptake related genes in the *C. hepaticus* isolate genomes after a DNA database was made with genes from iron uptake pathways in *C. jejuni* NCTC 11168 (Miller et al., 2009). The cut-offs were set at 80% for both identity and coverage so that the genes above these thresholds were recorded as present.

### Iron depletion assay

The growth of *C. hepaticus* isolates S11-010, S12-1018, and S12-0322 in regular Brain Heart Infusion broth (BHI) and in iron-depleted BHI was compared to that of *C. jejuni* NCTC 11168. *C. hepaticus* isolates were grown for 48 h on 5% sheep blood agar (SBA) plates at 42°C in a microaerobic atmosphere. NCTC 11168 was grown for 24 h under the same conditions. Growth was harvested into PBS at c. 105–106 cfu/ml and 100 μl added to 10 ml BHI in T25 tissue culture flasks. For each isolate, a regular broth and one containing the iron chelator deferoxamine mesylate (Desferal; Sigma) at a final concentration of 20 mM were inoculated (van Vliet et al., 1998). Each *C. hepaticus* isolate was tested in duplicate on 2 separate occasions, and NCTC 11168 was tested in duplicate on 4 separate occasions. Samples of the broths were removed at 1, 2, 3, and 5 days post-inoculation and quantitative bacteriology performed by plating out serial dilutions on to SBA plates (detection limit = 100 cfu/ml).

### Accession numbers

Nucleotide sequence data were submitted to the European Nucleotide Archive (http://www.ebi.ac.uk/ena) under the primary accession number PRJEB19094. Individual accession numbers are given in Table 1.

## Results

### *C. hepaticus* isolates form a distinct clade separate from other known *Campylobacter* species

**Figure 1:**
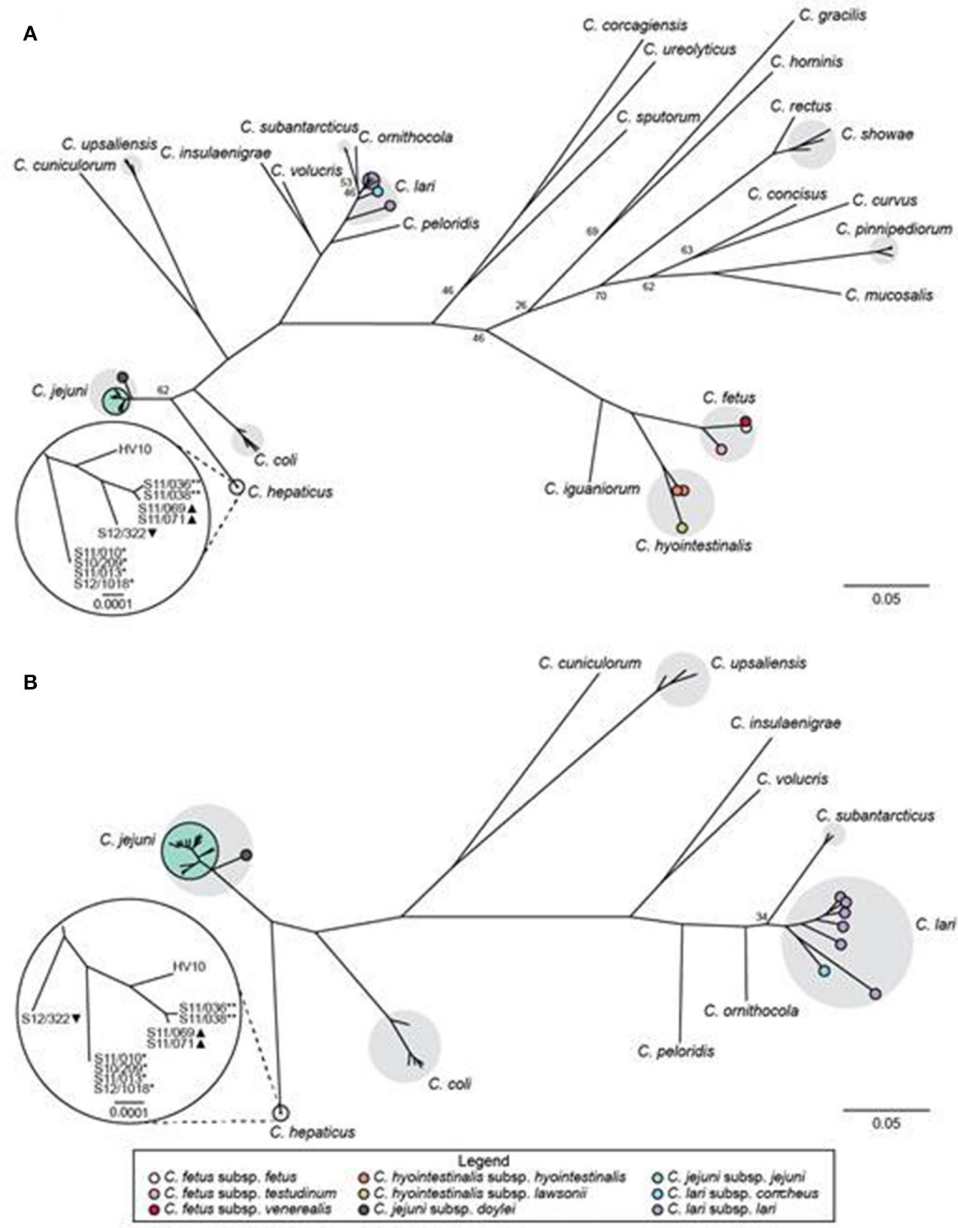
Relationships between *C. hepaticus* and other *Campylobacter* species based on gene-by-gene analyses. Maximum likelihood trees based on concatenated nucleotide sequences of **(A)** 52 rMLST genes from 86 isolates comprising 25 *Campylobacter* species; and **(B)** 646 core genes identified by cgMLST in ≥90% of 59 isolates comprising 11 closely related thermophilic *Campylobacter* species. Bootstrap values <80 **(A)** or <100 **(B)** are shown for major nodes. Gray shading indicates taxa with multiple representative isolates. Subspecies are marked with colored circles, as indicated in the legend. The insets provide magnified views of the relationships among *C. hepaticus* isolates. ^*^, farm 1; ^**^, farm 2; ▴, farm 4; ▾, farm 5.

We used a hierarchical gene-by-gene approach (Maiden et al., 2013) to investigate the relationships between the putative *C. hepaticus* isolates from the UK, the *C. hepaticus* type strain (HV10), and 24 other *Campylobacter* species. Complete nucleotide sequences of the 52 *rps* genes present in *Campylobacter* (Cody et al., 2013) were obtained from the 10 SLD-associated *Campylobacter* isolates sequenced for this study; however, isolate S12-0002 was contaminated and was excluded from further analyses, unless stated otherwise. In the rMLST phylogeny, the study isolates clustered most closely with HV10, confirming that they corresponded to *C. hepaticus*. The *C. hepaticus* cluster was positioned between *C. jejuni* and *C. coli* (Figure 1A). The *ad hoc* cgMLST comparison identified 646 genes that were present in ≥90% of 59 isolates including *C. hepaticus* and 10 other thermophilic *Campylobacter* species that clustered together in the rMLST phylogeny. The resulting cgMLST phylogeny was consistent with the rMLST tree, with *C. hepaticus* most closely related to *C. coli* (Figure 1B). Both phylogenies indicated that the UK *C. hepaticus* isolates were closely related and segregated according to farm (Figure 1).

### Local, farm-related phylogenetic clustering of *C. hepaticus* isolates

There was no *C. hepaticus* MLST scheme at the time of writing; therefore, we used a read-mapping approach to index variation at the 7 loci comprising the MLST scheme of *C. jejuni* and *C. coli*, the closest relatives to *C. hepaticus*. All study isolates, including S12-002, had identical *glnA, gltA*, and *pgm* alleles. There were 2 alleles each for *aspA, glyA, tkt*, and *uncA*, all of which corresponded to single nucleotide differences, except *uncA* which had 2 SNPs (Supplementary Figure S1). Although genetic diversity was low, SNPs were associated with sample origin: isolates from the same farm shared the same SNPs in the 7 core MLST genes (Supplementary Figure S1).

**Figure 2:**
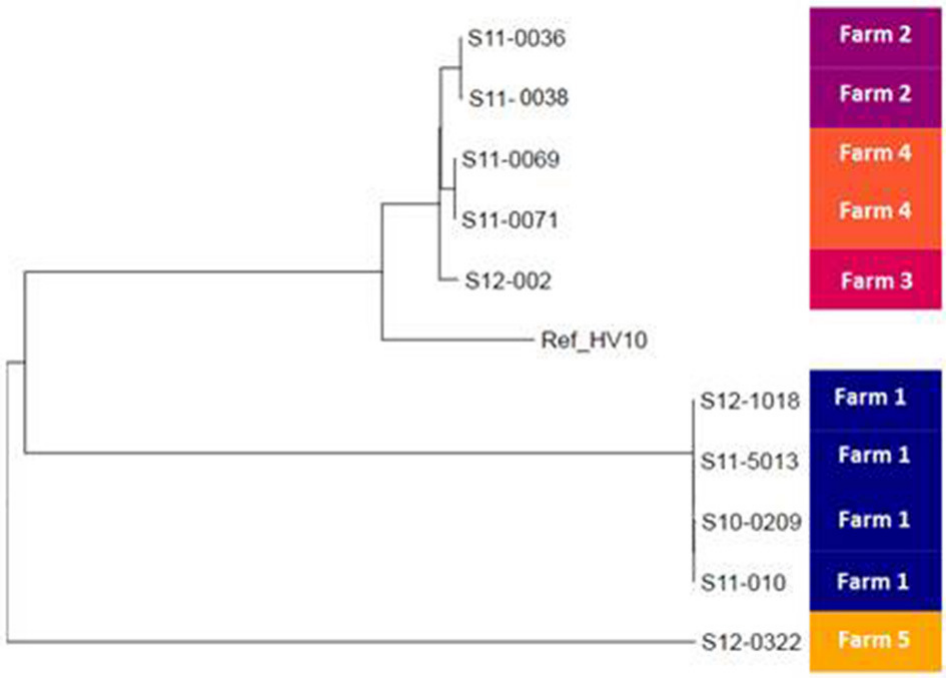
Phylogeny of the UK *Campylobacter hepaticus* isolates. Maximum likelihood tree constructed by reference to the whole genome sequence of isolate HV10. UK *C. hepaticus* isolates: S10-0209, S11-010, S11-5013, S12-1018, S11-0036, S11-0038, S12-002, S11-0071, and S12-0322.

To study the relationships among *C. hepaticus* isolates in more detail, a Maximum Likelihood phylogenetic tree was reconstructed using variable sites within the whole genome sequence with reference to the draft genome of HV10 (Figure 2). The contaminated isolate S12-002 was included in the mapping analyses. The phylogenetic tree also indicated clustering according to farm, with ≤ 11 SNP differences identified between the isolates collected from the same farm. Farm 1 isolates S10-0209, S11-010, S11-5013, and S12-1018 differed by a total of 5 SNPs (Figure 2). Similarly, isolates from farms 2 (S11-0036 and S11-0038) and 4 (S11-0069 and S11-0071) differed by 4 and 11 SNPs, respectively. In contrast, isolates from different farms were separated by at least an order of magnitude more SNPs, with HV10 clustering with isolates from farms 2, 3, and 4. Isolate S12-002 from farm 3 was 113 SNPs apart from S11-069 and S11-0071. Isolate S12-0322 from farm 5 was furthest apart from all other isolates in the phylogenetic tree, with 987 SNP differences to strains S11-0036 and S11-038. HV10 was 1161 SNPs apart from isolate S12-0322 (farm 5), 938 SNPs from S12-1018 (farm 1) and 614 SNPs from isolate S11-036 (farm 2; Figure 2).

### *C. hepaticus* isolates have reduced genomes

**Figure 3:**
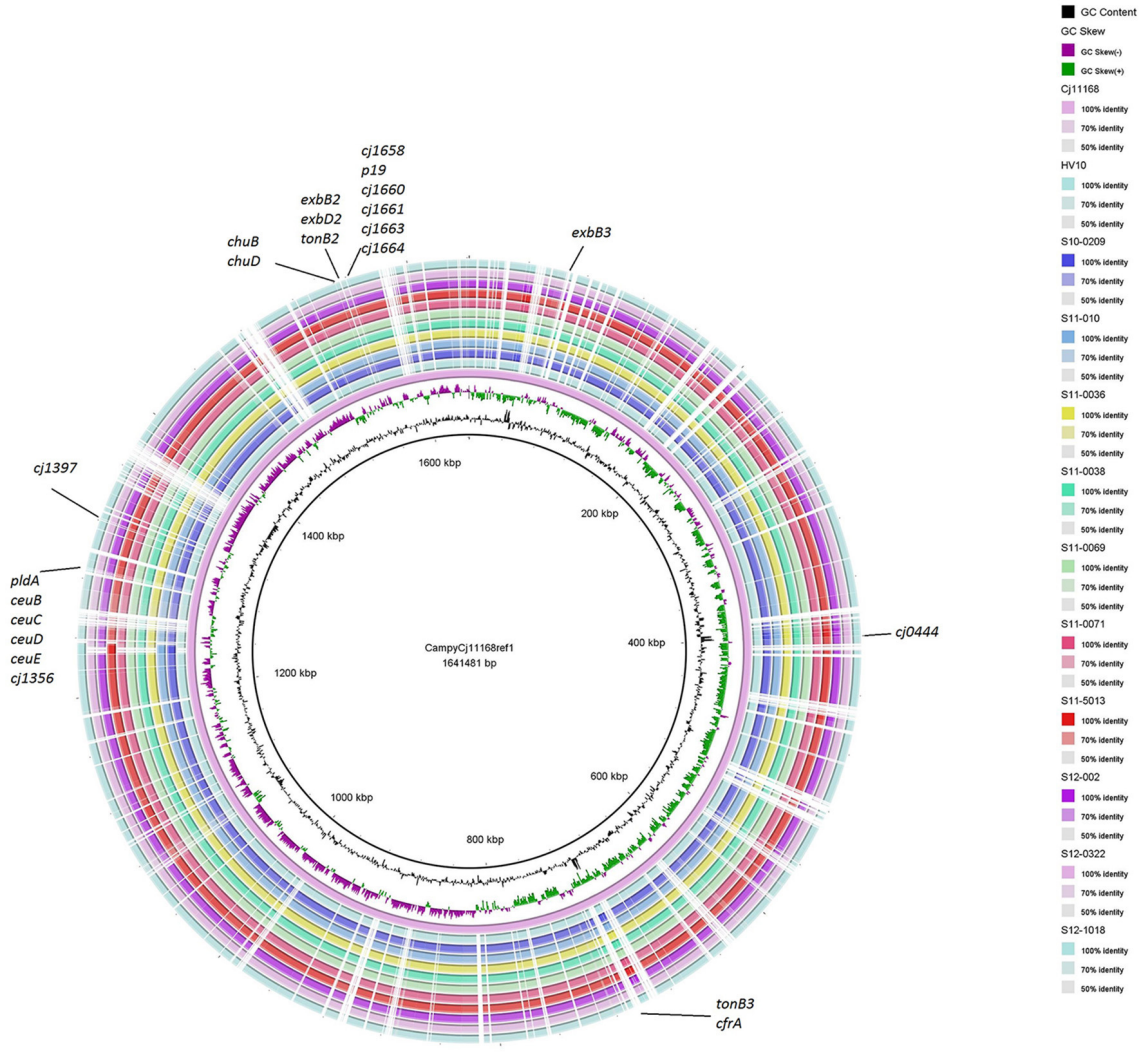
BRIG (BLAST Ring Image Generator) comparison of *C. hepaticus* isolates with *C. jejuni* NCTC 11168. Similarities between the reference genome *C. jejuni* NCTC 11168 and *C. hepaticus* isolates are presented as concentric rings. Inner circle: *C. jejuni* NCTC 11168 (purple ring). The 10 UK *C. hepaticus* isolates and HV10 are presented in the outer concentric rings.

The assembled contigs of the UK *C. hepaticus* genomes were submitted to RAST, Rapid Annotations based on Subsystem Technology, designed to annotate genes of prokaryotic genomes (Aziz et al., 2008). For comparison, the draft genome of HV10 (Van et al., 2016) and 5 *C. jejuni* genomes from the public databases, including NCTC 11168, M1, PT14, R14 and 4031 were also submitted to RAST, and pooled data of the UK *C. hepaticus* isolates were compared with the pooled data of the *C. jejuni* genomes (Table 1). The RAST results indicated that the UK *C. hepaticus* isolates were similar in size to each other, but had smaller genomes (1.53 Mb average) than the reference *C. jejuni* isolates, which were also similar in size (1.67 Mb average; *p* = 2.6 × E-4; Table 2). The reduction of ~140 Kb resulted in an average of 144 fewer genes (*p* = 6.1 × E-3). The *C. hepaticus* isolates had a lower number (average of 44) of RNA coding sequences (average of 52.4) and a lower GC content (average of 28.4%) in comparison to the *C. jejuni* reference genomes (average of 30.5%). The genome size of the Australian *C. hepaticus* HV10 isolate was 1.48 Mb with 27.9% GC (Table 2). Genome comparison using BLAST Ring Image Generator (BRIG) indicated multiple deletions in the 10 UK *C. hepaticus* isolates when compared to the *C. jejuni* NCTC 11168 genome (Figure 3).

**Table 2:** Comparison of *C. hepaticus* with *C. jejuni* (reference) genomes using RAST.

**RAST subsystems**	**HV-10**	**S10-0209**	**S11-010**	**S11-0036**	**S11-0038**	**S11-0069**	**S11-0071**	**S11-5013**	**S12-0322**	**S12-1018**	**Cj-M1**	**Cj-11168**	**Cj-PT14**	**Cj-R14**	**Cj-4031**	**Ave S**	**Ave R**	***t*-test**
Genome size (bp)	1482384	1549703	1596485	1498189	1501926	1490652	1516789	1551049	1538297	1530280	1616648	1641481	1635301	1795858	1669329	1530374	1671723	0.00
No. of genes	1512	1581	1629	1509	1511	1509	1513	1580	1531	1575	1610	1642	1631	1900	1677	1549	1692	0.01
No. of RNAs	46	42	44	44	45	44	47	44	45	44	53	53	50	53	53	44	52.4	0.00
CG content	27.9	28.5	28.5	28.4	28.4	28.1	28.5	28.5	28.4	28.2	30.6	30.5	30.5	30.4	30.5	28	30.5	0.00
Subsystem feature counts																		
Cofactors, vitamins, prosthetic groups, pigments	145	145	145	146	146	147	146	145	146	145	134	133	141	142	133	146	136.6	0.00
Cell wall and capsule	99	101	98	101	102	97	101	98	95	97	85	118	111	117	114	99	109	0.05
Virulence, disease, and defense	39	38	43	38	38	39	39	38	42	38	89	67	69	67	67	39	71.8	0.00
Potassium metabolism	8	8	8	8	8	8	8	8	8	8	12	13	12	13	12	8	12.4	0.00
Miscellaneous	4	4	4	4	4	4	4	5	4	4	4	4	4	4	4	4	4	0.48
Phages, prophages, transposons, plasmids	0	7	7	3	3	3	2	7	2	7	0	0	0	13	0	5	2.6	0.38
Membrane transport	47	48	59	48	48	48	48	48	66	48	41	45	45	49	40	51	44	0.05
Iron acquisition and metabolism	5	5	5	5	5	5	5	5	5	5	45	50	50	41	46	5	46.4	0.00
RNA metabolism	68	68	68	68	68	68	68	68	68	68	70	71	70	70	71	68	70.4	0.00
Nucleosides and nucleotides	48	47	47	47	47	48	47	47	47	47	50	50	50	50	50	47	50	0.00
Protein metabolism	212	210	211	211	211	210	210	210	213	210	215	215	214	214	215	211	214.6	0.00
Cell division and cell cycle	9	9	10	9	9	9	9	9	10	9	22	22	22	22	22	9	22	0.00
Motility and chemotaxis	81	83	83	81	81	83	82	83	82	83	83	85	84	85	84	82	84.2	0.00
Regulation and cell signaling	20	20	21	21	20	20	20	21	21	20	20	17	17	20	20	20	18.8	0.02
DNA metabolism	40	41	46	42	40	42	42	41	47	41	50	52	52	50	50	42	50.8	0.00
Fatty acids, lipids, and isoprenoids	71	79	79	72	72	72	72	79	69	79	63	64	64	77	69	75	67.4	0.02
Nitrogen metabolism	14	17	17	14	14	13	13	17	14	17	12	13	14	13	12	15	12.8	0.02
Respiration	78	77	77	77	77	77	77	77	80	77	72	71	72	72	73	77	72	0.00
Stress response	36	36	36	36	36	36	36	36	36	36	41	43	43	43	43	36	42.6	0.00
Amino acids and derivatives	245	251	251	244	244	241	241	251	241	251	280	286	284	293	284	246	285.4	0.00
Carbohydrates	83	112	112	84	84	86	86	112	82	112	62	62	62	75	68	97	65.8	0.00

*C. hepaticus isolates: draft Australian C. hepaticus genome HV10. UK isolates: S10-0209, S11-010, S11-0036, S11-0038, S11-5013, S12-0322, and S12-1018. Reference genomes: C. jejuni NCTC 11168, M1, PT14, R14, and 4031*.

### Functional annotation

**Figure 4:**
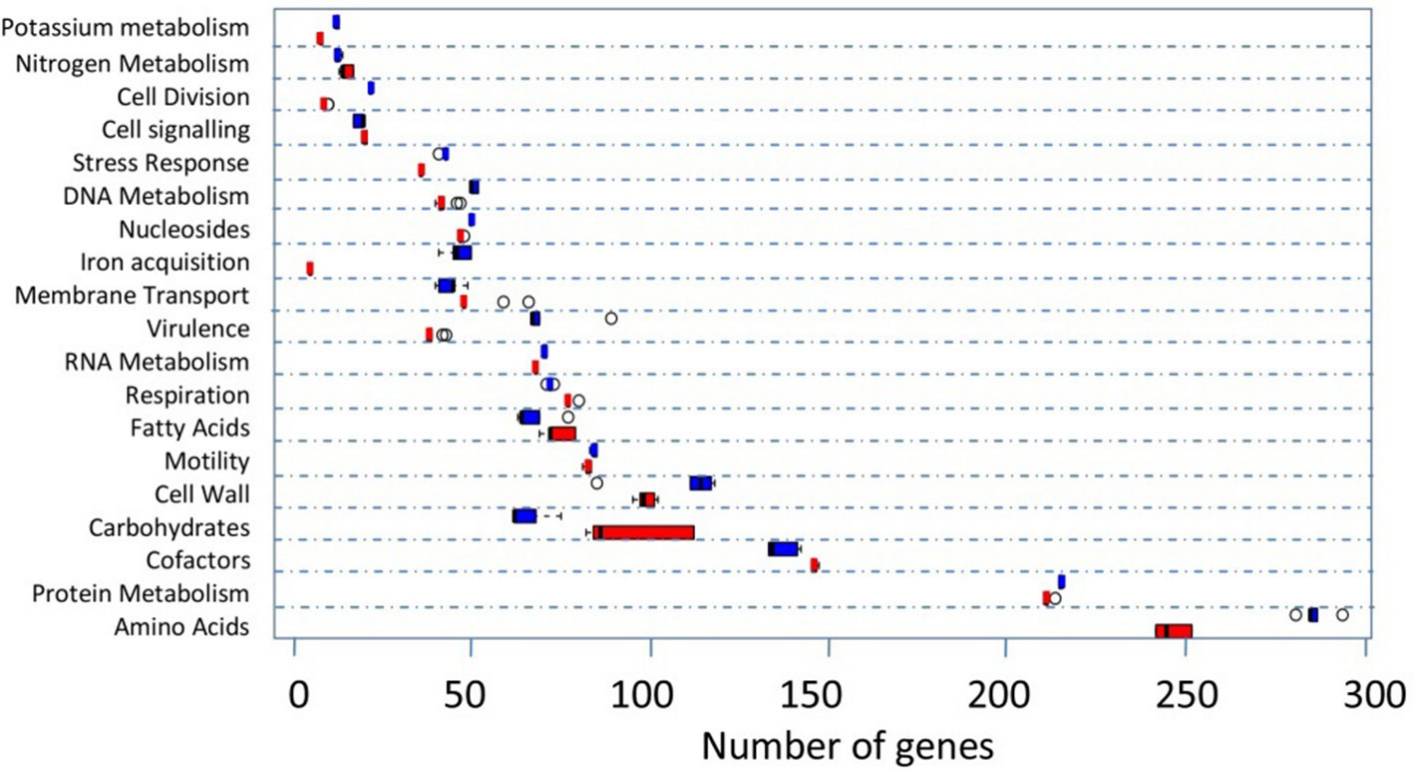
Comparison of the functional subsystems in the UK *C. hepaticus* (red) and 5 *C. jejuni* reference genomes (blue) as generated by RAST. References: M1, NCTC 11168, PT14, R14, and 4031. The open circles represent the outliers. Only subsystems with significant differences (student's *t*-test) are shown. The names of some subsystems are shortened: Cell Division, Cell Division and Cell Cycle; Cell signaling, Regulation and Cell signaling; Nucleosides, Nucleosides and Nucleotides; Iron acquisition, Iron acquisition and metabolism; Virulence, Virulence, Disease and Defense; Fatty Acids, Fatty Acids, Lipids, and Isoprenoids; Motility, Motility and Chemotaxis; Cell Wall, Cell Wall and Capsule; Cofactors, Cofactors, Vitamins, Prosthetic Groups, Pigments; Amino Acids, Amino Acids and Derivatives.

**Figure 5:**
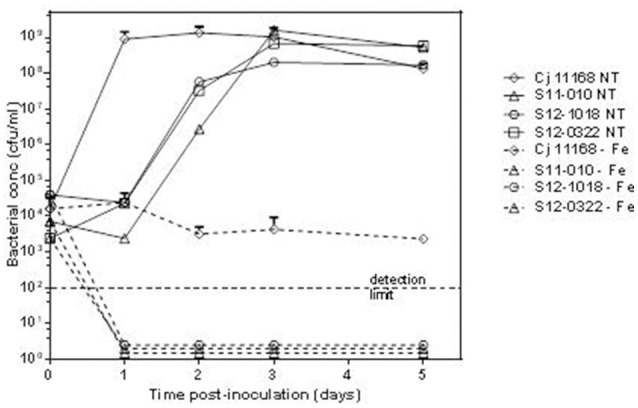
Reduced growth of *C. hepaticus* isolates in iron depleted conditions. Growth curves for 3 UK *C. hepaticus* isolates and *C. jejuni* NCTC 11168 grown in Brain Heart Infusion broth either regular or untreated (NT) or iron-depleted (−Fe).

RAST uses FIGfam (Aziz et al., 2008) to cluster annotated genomes in subsystems that are further divided into groups, based on protein sequence similarity. Clustered genes are listed as hierarchical subsystems that display increasing functional extensiveness (Overbeek et al., 2005; Table 2). Nineteen of the 21 subsystems reached statistical significance when pooled UK *C. hepaticus* isolates were compared to pooled *C. jejuni* reference genomes (Figure 3). *C. hepaticus* genomes contained significantly fewer genes than the *C. jejuni* references in 11 of the 21 clustered subsystems, and significantly more in 8 subsystems (Figure 4 and Table 2). The largest decrease was in the subsystem containing genes for iron acquisition and metabolism, with the *C. hepaticus* isolates containing on average only five genes in comparison to the average of 46.4 (or 11%) present in the reference genomes. Furthermore, within this subsystem, there were no genes identified in the group for iron transport in *C. hepaticus*, while 8 related genes were identified in *C. jejuni*. To confirm the absence of iron uptake genes in *C. hepaticus*, the study genomes were searched using blastn to identify genes from the *C. jejuni* NCTC 11168 iron uptake pathways (Miller et al., 2009). The cut-offs were set at 80% for both identity and coverage so that the genes above these thresholds were recorded as present. The following loci could not be detected among the UK *C. hepaticus* isolates: 7/8 genes from the ferri-enterochelin pathway; the entire ferri-rhodotorulic acid pathway; 5/8 genes in the haem pathway; 1/2 genes in the ferrous iron pathway; and cj0444 was missing from the cj0444 pathway (Supplementary Figure S2). The loss of function was tested with an iron depletion assay. There were clear differences between the growth of the *C. hepaticus* isolates and *C. jejuni* NCTC 11168 (Figure 5). In regular broth, the *C. hepaticus* isolates reached peak levels (108–109 cfu/ml) at 3 days post-inoculation, compared to only 1 day for *C. jejuni*. In the iron-depleted media, the *C. jejuni* persisted for 5 days at approximately starting levels (103–104 cfu/ml). In contrast, there were no detectable colonies on agar plates at any time point after adding the iron chelator in all tested *C. hepaticus* isolates.

There were fewer putative virulence, disease, and defense subsystem genes in the UK *C. hepaticus* isolates, with an average of 39.2 genes identified compared with 71.8 in the *C. jejuni* genomes. Within this subsystem, on average 13 genes associated with adhesion (adhesion subgroup) were present in the reference genomes, but no known adhesion genes were identified in the *C. hepaticus* isolates. Similarly, there were six genes in the cytolethal distending toxin (CDT) group in the reference genomes, but no genes of this group were present in the *C. hepaticus* genomes. In the resistance to antibiotics and toxic compounds subgroup there were 3–5 arsenic resistance genes in the reference genomes, but no resistance genes were present in the *C. hepaticus* chromosomes.

In the DNA metabolism group, the reference genomes typically contained 5–6 type I restriction-modification pathways; the *C. hepaticus* isolates contained one of these pathways. On average, 36 genes for stress response were found in the *C. hepaticus* genomes, which was significantly lower than the 42.6 genes of this group present in the reference genomes. In the oxidative stress pathway, each of the reference genomes contained four genes in the redox-dependent regulation of nucleus processes subsystem and 2 genes in the rubrerythrin subsystem, all of which were absent in the *C. hepaticus* genomes.

In the amino acids and derivatives group, the pathways of arginine, urea cycle, and polyamines differed between the UK *C. hepaticus* isolates and reference genomes. The *C. hepaticus* genomes typically had: putrescine utilization pathways (two genes) that were absent in the reference genomes; a lower number of genes in the arginine deiminase pathway (16 in comparison to 29 in the reference genome); a lower number of polyamine metabolism genes (21 in comparison to 32); and fewer arginine and ornithine degradation genes (20 in comparison to 33 in reference genomes).

The carbohydrates and fatty acids, lipids, and isoprenoids groups were among the 8 subsystems with significantly more genes in the UK *C. hepaticus* isolates than the *C. jejuni* reference genomes. The *C. hepaticus* isolates contained on average 96.7 genes in the carbohydrates group, while an average of only 65.8 genes were present in the reference genomes (Supplementary Table S2).

### Genes related to the pathogenicity of *C. hepaticus*

The genomes of HV10 and the UK *C. hepaticus* isolates, including S12-002, and *C. jejuni* isolates NCTC 11168, M1 and 4031, were submitted to the PathogenFinder database (v 1.1) (https://cge.cbs.dtu.dk/services/PathogenFinder) to identify the presence of pathogenicity-related genes by comparison of the protein families of all bacterial pathogens present in the database. A total of 511 pathogenicity related factors were identified in the genomes of NCTC 11168, 372 in M1 and 342 in 4031, while none was found in HV10. The UK *C. hepaticus* isolates contained relatively few genes linked to pathogenesis: 5 were identified in the genomes of S11-0069, S11-0071, S11-0038, S11-0036, and S12-002 (from farms 2, 3, and 4); 6 in S11-0038 (farm 2); 15 in S10-0209, S11-010, S12-1018, S11-5013 (farm1); and 7 in isolate S12-0322 (farm 5; Table 3). The *cpp* and *cmgB3/4* genes, both components of the pTet plasmid (Batchelor et al., 2004), were identified in isolates S11-10, S12-0322, and S12-002 (Table 3). Complete pTet plasmid (Batchelor et al., 2004) sequences were identified in isolates S11-010, S12-002, and S12-0322.

**Table 3:** Presence of pathogenicity-related genes in *C. hepaticus*.

**Protein (name)**	**Protein ID**	**S11-0036 F2**	**S11-0038 F2**	**S11-0069 F4**	**S12-0071 F4**	**S12-0209 F1**	**S11-1018 F1**	**S10-5013 F1**	**S12-010 F1**	**S12-0322 F5**	**S12-002 F3**
MCP	EAQ73158										
TrkA	ABS44147										
CHP1	EAQ72353										
CHP2	EAQ72298										
HP1	EAQ71971										
HAD-superfamily phosphatase, subfamily IIIC	EAQ72583										
Putative 3-oxoacyl- synthase	ABS43995										
Methyltransferase	CAL35414										
DNA adenine methylase	AAW34814										
HP2	EAQ72552										
HP3	HP3										
Putative DNA-binding protein	AAW34848										
Putative acyl carrier protein	CAL35413										
Putative acyl carrier protein	AAW35934										
CHP3	EAQ71755										
Putative SAM domain containing methyltransferase	CAL35414										
CHP4	EAQ72353										
cpp14	AAR29498.1										
cpp17	AAR29501.										
cpp22	AAR29505.										
cpp18	AAR29502.										
cpp47	AAR29530.										
cpp45	AAR29528.										
cpp29	AAR29512.										
cpp13	AAR29497.1										
pTet	AY714214.										
cmgB3/4	AAR29514.1										

*Purple, present; blank, absent; orange, plasmid pTet (pCC31, AY394560.1) related proteins; dark blue, proteins not present in C. jejuni 11168. Farms 1–5 are indicated (F1–F5)*.

Five genes related to pathogenicity in *Campylobacter* spp. were present in *C. hepaticus* but not in *C. jejuni* isolates NCTC 11168, M1 and 4031: (1) putative potassium uptake protein TrkA (ABS44147) identified in *C. jejuni* subsp. *doylei* 269.97 (PRJNA17163); (2) HAD-superfamily phosphatase, subfamily IIIC protein EAQ72583; (3) DNA adenine methylase protein AAW34814 identified in *C. jejuni* RM1221; (4) Hypothetical HP2 protein EAQ72552; and (5) CHP3 conserved hypothetical protein EAQ71755 (Table 3).

## Discussion

*C. hepaticus* has been identified as the cause of SLD (Crawshaw et al., 2015; Van et al., 2016, 2017), and the disease pathology reproduced in SPF birds in the UK and mature layer hens in Australia; however, our understanding of the genomics and evolution of this emerging pathogen remain limited. Hierarchical gene-by-gene analyses of putative *C. hepaticus* isolates from SLD cases in the UK and representatives of 25 *Campylobacter* species confirmed that the UK isolates were most closely related to HV10, the *C. hepaticus* type strain. This verified that *C. hepaticus* is a cause of SLD in both the UK and Australia, as hypothesized by Van et al. (2016). Previous studies suggested that *C. hepaticus* was most closely related to members of the *C. lari* group or *C. jejuni* and *C. coli* (Crawshaw et al., 2015; Van et al., 2016); however, these findings were based on phylogenetic analyses of 16S rRNA or heat shock protein 60 gene sequences. The limitations of single gene phylogenies for inferring relationships among species have been acknowledged, particularly 16S rRNA gene sequencing for *Campylobacter* taxonomy (Gorkiewicz et al., 2003; Miller et al., 2012, 2014b). The higher resolution rMLST and cgMLST analyses carried out in this study confirmed that *C. hepaticus* was positioned between the major human pathogens *C. jejuni* and *C. coli*, which clustered with *C. upsaliensis, C. cuniculorum*, and members of the *C. lari* group. These are all thermotolerant spp., many of which have been isolated from birds and some corresponding to emerging human pathogens (Kaakoush et al., 2015).

When analyzed at the MLST, rMLST, and cgMLST levels, the UK *C. hepaticus* isolates were highly similar to each other. Isolates from farms 2, 3, and 4 all shared the same MLST profile, while those from farms 1 and 5 differed at one and two loci, respectively, including just 5 SNPs in total; none of these profiles appeared in the *C. jejuni/coli* PubMLST database. At the rMLST and cgMLST levels, the UK isolates remained highly similar, but clustered by farm. High-resolution SNP analysis was used to resolve the relationships among the study isolates, revealing low levels of within-farm diversity. Isolates from the same farm differed by 3–12 SNPs, which contrasted with higher levels of between-farm diversity (173–1,260 SNPs). That the highly similar farm 1 isolates were collected between 2010 and 2012 suggests that these genotypes are stable over time. Overall, the low levels of within-farm diversity were similar to those observed in campylobacteriosis outbreaks (Llarena and Taboada, 2017). Although, the sample size was small, the clustering of isolates suggested a farm-specific subpopulation structure that may reflect ongoing local microevolution, while the between-farm diversity indicated that *C. hepaticus* is not a newly emerged pathogen. It was of interest that the Australian isolate HV10 was positioned within the diversity of the UK *C. hepaticus* isolates. Further sampling will be necessary to fully characterize the population structure and global epidemiology of *C. hepaticus*. When genome sequencing is not feasible, a new MLST scheme based on the *C. jejuni/coli* scheme may prove beneficial.

Reductive genome evolution has been described in diverse bacteria and is typically associated with specialization, often in an intracellular niche (Georgiades and Raoult, 2010; McCutcheon and Moran, 2011). At 1.48 Mbp, HV10 is the smallest *Campylobacter* genome sequenced to date (Supplementary Table S1), while the UK *C. hepaticus* isolates had a slightly larger average genome size of 1.53 Mbp. This represents a reduction of ~171–238 kb compared to their closest relatives *C. jejuni* and *C. coli*, which also have relatively small genomes compared to other *Campylobacter* species (Supplementary Table S1 and references therein). RAST annotation of the UK *C. hepaticus* genomes indicated a reduction of ~144 genes and 8 RNA coding sequences compared to five *C. jejuni* reference genomes. Large-scale gene loss and inactivation have been reported in several niche-adapted bacterial pathogens, including: *Shigella* spp. (Maurelli et al., 1998; Wei et al., 2003); *Mycobacterium leprae* and *Mycobacterium ulcerans* (Cole et al., 2001; Rondini et al., 2007); *Bordetella pertussis* and *Bordetella parapertussis* (Parkhill et al., 2003); and *Rickettsia* spp. (Merhej and Raoult, 2011). Likewise, a study of bacteria with different lifestyles identified fewer genes involved in transcription and translation in obligate intracellular bacteria (Merhej et al., 2009). Reduced bacterial genomes also tend to shift toward a higher AT content (McCutcheon and Moran, 2011). The *C. hepaticus* isolates had a lower average GC content (28.4%) than *C. jejuni* (30.5%) and most other *Campylobacter* species (Supplementary Table S1). Likely drivers of the genome reduction observed in *C. hepaticus* include specialization and genetic isolation following the occupation of a new niche (Georgiades and Raoult, 2010), namely the chicken liver, and perhaps also the transition from a free-living or facultatively parasitic life-cycle to an obligate pathogenic life-cycle (Moran, 2002).

Genome reduction results in gene losses across all functional categories, with biosynthetic pathways commonly eliminated when metabolites are available from the environment (Toft and Andersson, 2010; McCutcheon and Moran, 2011; Hottes et al., 2013; Albalat and Canestro, 2016). In *C. hepaticus*, 11 out of 21 subsystems defined by RAST were reduced compared to *C. jejuni*. Gene loss was particularly evident among iron metabolism pathways in *C. hepaticus*, consistent with adaptation to an iron rich environment such as the chicken liver. The *C. hepaticus* isolates contained only 10% of the iron metabolism related genes present in *C. jejuni* isolates (Supplementary Figure S2). Eight subsystems were identified with a higher number of genes in the *C. hepaticus* isolates than in the reference *C. jejuni* genomes (Figure 4) with the highest number of gene differences in carbohydrate utilization pathways (average of 96.7 genes in the study isolates and 65.8 in *C. jejuni*; Supplementary Table S2). This is interesting as *Campylobacter* is generally considered to be a non-saccharolytic bacterium unable to use glucose and other carbohydrate sources as a growth substrate (Hofreuter, 2014), an observation supported by WGS and BIOLOG studies (Parkhill et al., 2000; Bochner, 2009; Gripp et al., 2011). Carbon source utilization is characteristic for growth of other intracellular gastrointestinal pathogens, for instance *Salmonella* Typhimurium and *Listeria monocytogenes* (Dandekar et al., 2012; Fuchs et al., 2012), as well as the close relative of *Campylobacter, Helicobacter pylori* (Mendz et al., 1993). However, recent studies demonstrated that some *C. jejuni* strains can metabolize the sugar L-fucose due to the presence of a novel L-fucose pathway including L-fucose permease within a 9 kb genomic island in these strains (Muraoka and Zhang, 2011; Stahl et al., 2011). Furthermore, Stahl and co-workers found that the ability to meatbolise L-fucose *in vivo* provided *C. jejuni* with competitive advantage during colonization of the piglet infection model. Similar was not observed in the chick commensal model (Stahl et al., 2011), suggesting potential niche specific advantage for colonization in L-fucose reach environment in the pig small intestine and cecum. It is possible that the *C. hepaticus* have adopted different carbohydrate utilization mechanisms for opportunistic growth in a carbohydrate rich intracellular environment in the chicken liver.

Reduced genomes can be associated with niche adaptation and increased pathogenicity in some bacteria (Moran, 2002). Niche adaptation requires selection for and against traits to optimize pathogen fitness in the new environment (Bliven and Maurelli, 2012). In *C. hepaticus*, there was a large reduction in “virulence factors,” with only 5–15 recognized pathogenicity genes detected in these isolates. In contrast, *C. jejuni* isolates NCTC 11186, M1 and 4031 contained 511, 372, and 342 pathogenicity genes, respectively. With respect to pathogenicity factors identified in *C. hepaticus*, TrkA, a homolog of the putative potassium uptake protein described as an essential protein for maintenance of ionic homeostasis in response to changes in the environment (Lee et al., 2007), was present in all study isolates but not all *C. jejuni* reference genomes. The same was true of four other genes: a homolog of the two-component system methyl-accepting chemotaxis proteins (MCPs) that serve as sensors in bacterial chemotactic signaling, detecting attractants, and promoting bacterial movement toward suitable sites for colonization (Li et al., 2014); and 3 conserved hypothetical proteins CHP1, CHP2, and HP1, which have been described in *C. jejuni* 81–176. Interestingly, a subset of *C. hepaticus* isolates also contained a homolog protein that is part of the haloacid dehydrogenase (HAD) superfamily, which are involved in a variety of cellular processes ranging from amino acid biosynthesis to detoxification and has only been described in strain 81–176. Similarly, the hypothetical protein HP2 and the conserved hypothetical protein CHP3 present in some *C. hepaticus* isolates have also only been previously described in strain 81–176. Strain 81–176 displays increased virulence and invades intestinal epithelial cells at levels that are as much as 3 logs higher than other invasive *C. jejuni* strains (Poly et al., 2005). Furthermore, there was a large reduction in the genes encoding capsular and extracellular polysaccharides (CPS); CPS produced by *C. jejuni* are known to be important virulence factors that are involved in colonization and invasion (Richards et al., 2013). There were also fewer putative virulence, disease, and defense subsystem genes in the *C. hepaticus* isolates, including the absence of the CDT group genes encoding a bacterial toxin that initiates a eukaryotic cell cycle block at the G2 stage prior to mitosis (Jinadasa et al., 2011). It is possible that the evolution of attenuated virulence in *C. hepaticus* could have occurred as a result of immune evasion within the host (Mikonranta et al., 2015) that enables potential establishment of a long term chronic infection (Dennis, 2016) in laying hens with disease manifestation around pick lay. Further analyses of a larger, global *C. hepaticus* isolate collection are required to robustly infer the pan-genome of *C. hepaticus*, which in turn will improve our understanding of niche specialization in this organism.

This work highlights the potential importance of *C. hepaticus* to the poultry industry, especially as infection is likely to be under-detected because isolation requires modifications to the standard *C. jejuni/C. coli* protocol. Further work is needed to improve the sampling and isolation methods for detection of *C. hepaticus* on poultry farms. *C. hepaticus* has not yet been reported in humans; however, consumption of chicken liver is a common source of campylobacteriosis outbreaks (Noormohamed and Fakhr, 2012; Weber et al., 2014; Moffatt et al., 2016). Detection of the *C. jejuni* pTet tetracycline resistance plasmid in 3 study isolates from 3 separate farms is also a cause of concern. Transfer of genetic material between *C. hepaticus* and other *Campylobacter* spp. may mediate exchange of antimicrobial resistance and pathogenicity-related determinants. Further studies of additional isolates are necessary to better understand the population structure and evolution of this important pathogen.

## Author contributions

LP, TC, and RI designed the study; LP, YT, MJvR performed the analyses; JN and RE helped with the analyses; MJvR, TC, AW, SC, and SS revised the manuscript and provided valuable suggestions and LP wrote the manuscript.

### Conflict of interest statement

The authors declare that the research was conducted in the absence of any commercial or financial relationships that could be construed as a potential conflict of interest.

## Supplementary material

Supplementary Figure S1 MLST diversity among UK *C. hepaticus* isolates. Nucleotide sequence alignments of the 7 gene fragments comprising the *C. jejuni*/*coli* MLST scheme are shown. Consensus sequences were determined by mapping raw reads to the sequence of S12-1018. Variable sites are marked with arrows.

Supplementary Figure S2 Comparison of *C. hepaticus* and *C. jejuni* iron uptake pathways. The figure was adapted from a review by Miller et al. (2009). Iron uptake genes in the *C. jejuni* NCTC 11168 genome were used to make a database and UK *C. hepaticus* genomes were queried using blastn with 80% cut-offs for both identity and coverage. Blue arrows: iron uptake related genes with arrowheads indicating the direction of transcription; white arrows: flanking genes. Red: genes not detected in *C. hepaticus*.

Supplementary Table S1 Isolates used in this study.

Supplementary Table S2 Comparison of the Carbohydrates subsystem in *C. hepaticus* and *C. jejuni* (reference) genomes using RAST.
