# Corrigendum: Genome Reduction for Niche Association in *Campylobacter Hepaticus*, A Cause of Spotty Liver Disease in Poultry

**DOI:** 10.3389/fcimb.2017.00480

**Published:** 2017-11-14

**Authors:** Liljana Petrovska, Yue Tang, Melissa J. Jansen van Rensburg, Shaun Cawthraw, Javier Nunez, Samuel K. Sheppard, Richard J. Ellis, Adrian M. Whatmore, Tim R. Crawshaw, Richard M. Irvine

**Affiliations:** ^1^Bacteriology, Animal and Plant Health Agency Weybridge, Addlestone, United Kingdom; ^2^Department of Zoology, University of Oxford, Oxford, United Kingdom; ^3^NIHR Health Protection Research Unit in Gastrointestinal Infections, University of Oxford, Oxford, United Kingdom; ^4^Veterinary Surveillance, Animal and Plant Health Agency Weybridge, Addlestone, United Kingdom; ^5^Department of Biology and Biotechnology, The Milner Centre for Evolution, University of Bath, Bath, United Kingdom

**Keywords:** *Campylobacter hepaticus*, spotty liver disease, poultry, genome reduction, niche adaptation

In the original article, there was a mistake in Table 3 as published. Table 3 had additional genes inserted for isolates S11-0036, S11-0038, S11-0069, and S12-0071. Isolate S12-002 should not be included in Table 3.

**Table 3:** Presence of pathogenicity-related genes in *C. hepaticus*.

**Protein (name)**	**Protein ID**	**S11-0036**	**S11-0038**	**S11-0069**	**S11-0071**	**S10-0209**	**S12-1018**	**S11-5013**	**S11-010**	**S12-0322**
		**F2**	**F2**	**F4**	**F4**	**F1**	**F1**	**F1**	**F1**	**F5**
MCP	EAQ73158									
TrkA	ABS44147									
CHP1	EAQ72353									
CHP2	EAQ72298									
HP1	EAQ71971									
HAD-superfamily phosphatase, subfamily IIIC	EAQ72583									
Putative 3-oxoacyl- synthase	ABS43995									
Methyltransferase	CAL35414									
DNA adenine methylase	AAW34814									
HP2	EAQ72552									
HP3	HP3									
Putative DNA-binding protein	AAW34848									
Putative acyl carrier protein	CAL35413									
Putative acyl carrier protein	AAW35934									
CHP3	EAQ71755									
Putative SAM domain containing methyltransferase	CAL35414									
CHP4	EAQ72353									
cpp14	AAR29498.1									
cpp17	AAR29501.									
cpp22	AAR29505.									
cpp18	AAR29502.									
cpp47	AAR29530.									
cpp45	AAR29528.									
cpp29	AAR29512.									
cpp13	AAR29497.1									
pTet	AY714214.									
cmgB3/4	AAR29514.1									

*Purple, present; blank, absent; orange, plasmid pTet (pCC31, AY394560.1) related proteins, dark blue, proteins not present in C. jejuni 11168. Farms 1, 2, 4, and 5 are indicated (F1, F2, F4, and F5)*.

Additionally, there was an incorrect sentence. Incorrect sentence describing the number of RNA coding sequences and the GC content. A correction has been made to Results, *C. hepaticus* Isolates Have Reduced Genomes, Paragraph Number One and appears below.

The *C. hepaticus* isolates had a lower number (average of 44) of RNA coding sequences and a lower GC content (average of 28.4%) in comparison to the *C. jejuni* reference genomes (average of 52.4 and 30.5%, respectively).

Similarly, there was an incorrect sentence. Incorrect sentence describing the genes related to pathogenicity of *C. hepaticus*. A correction has been made to Results, genes related to the Pathogenicity of *C. hepaticus*, Paragraph Number One and appears below.

The UK *C. hepaticus* isolates contained relatively few genes linked to pathogenesis: 5 were identified in the genomes of S11-0036, S11-0069, S11-0071 and (from farms 2, and 4); 6 in S11-0038 (farm 2); 15 in S10-0209, S12-1018, S11-5013, and S11-010, (farm 1); and 7 in isolate S12-0322 (farm 5; Table 3). The *cpp* and *cmgB3/4* genes, both components of the pTet plasmid (Batchelor et al., 2004), and a complete pTet plasmid (Batchelor et al., 2004) sequences were identified in isolates S11-010, and S12-0322 (Table 3).

Finally, in incorrect spelling of metabolism was used, we omitted “the” and misspelled “rich.” A correction has been made to Discussion, Paragraph Number Four and appears below.

Furthermore, Stahl and co-workers found that the ability to metabolize L-fucose *in vivo* provided *C. jejuni* with competitive advantage during colonization of the piglet infection model. Similar was not observed in the chick commensal model (Stahl et al., 2011), suggesting potential niche specific advantage for colonization in the L-fucose rich environment in the pig small intestine and cecum.

The authors apologize for these errors and state that this does not change the scientific conclusions of the article in any way.

## Conflict of interest statement

The authors declare that the research was conducted in the absence of any commercial or financial relationships that could be construed as a potential conflict of interest.

