# Supplementary figures and images for: Genome Reduction for Niche Association in Campylobacter Hepaticus, A Cause of Spotty Liver Disease in Poultry
Source: Front Cell Infect Microbiol. 2017 Aug 11;7:354. doi: 10.3389/fcimb.2017.00354 (PMC5554493; doi:10.3389/fcimb.2017.00354)

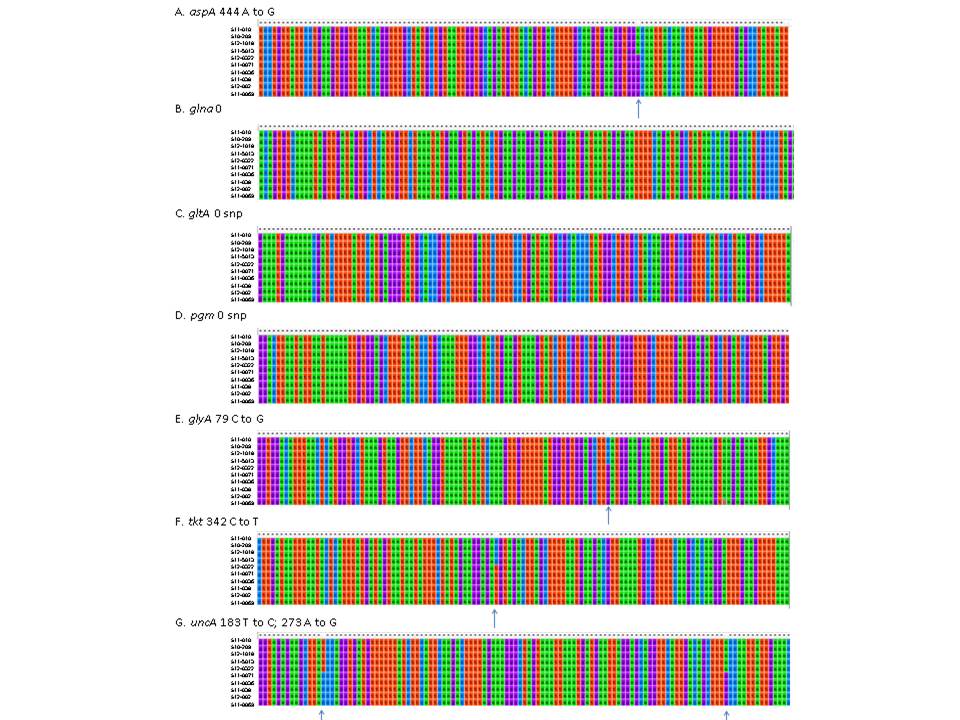

Supplement: Supplementary Figure S1 — MLST diversity among UK C. hepaticus isolates. Nucleotide sequence alignments of the 7 gene fragments comprising the C. jejuni/coli MLST scheme are shown. Consensus sequences were determined by mapping raw reads to the sequence of S12-1018. Variable sites are marked with arrows. [file Image1.JPEG]

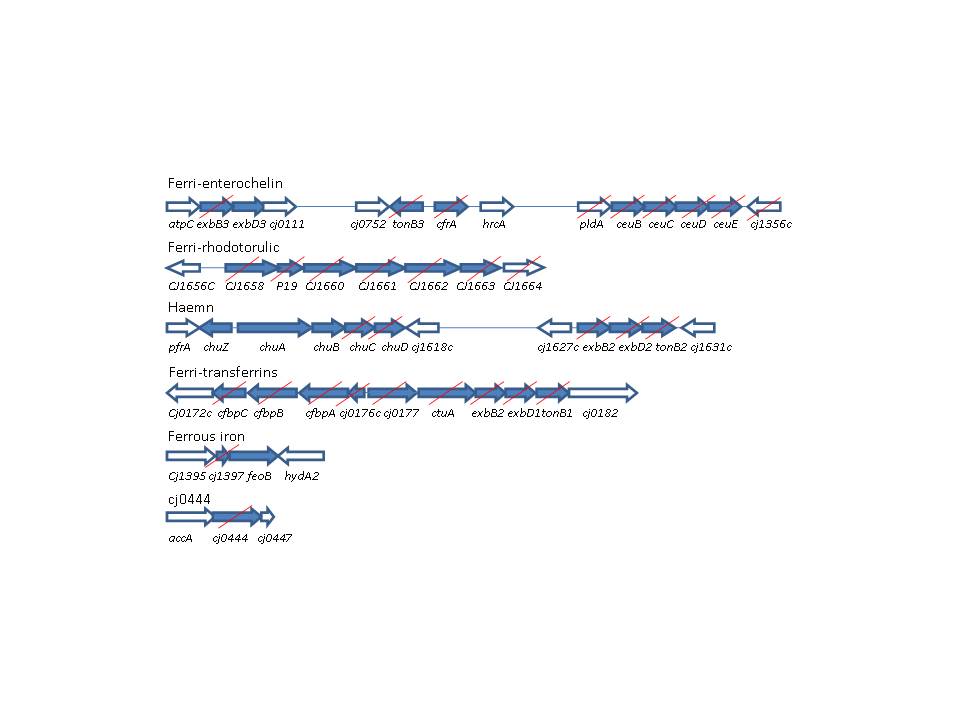

Supplement: Supplementary Figure S2 — Comparison of C. hepaticus and C. jejuni iron uptake pathways. The figure was adapted from a review by Miller et al. (2009). Iron uptake genes in the C. jejuni NCTC 11168 genome were used to make a database and UK C. hepaticus genomes were queried using blastn with 80% cut-offs for both identity and coverage. Blue arrows: iron uptake related genes with arrowheads indicating the direction of transcription; white arrows: flanking genes. Red: genes not detected in C. hepaticus. [file Image2.jpeg]
