# Supplementary material for: Genome Reduction for Niche Association in Campylobacter Hepaticus, A Cause of Spotty Liver Disease in Poultry
Source: Front Cell Infect Microbiol. 2017 Aug 11;7:354. doi: 10.3389/fcimb.2017.00354 (PMC5554493; doi:10.3389/fcimb.2017.00354)
Supplement: Supplementary Table S1 — Isolates used in this study. [file Table1.DOC]

Table S1. Isolates used in this study

| **Species** | **Isolate** | **GenBank accession number** | **Size (Mb)** | **% GC** | **References** |
| --- | --- | --- | --- | --- | --- |
| *C. coli* | RM1875 | CP007183.1 | 1.86 | 31.1 | - |
|  | RM4661 | CP007181.1 | 1.87 | 31.1 | - |
|  | FB1 | CP011015.1 | 1.73 | 31.4 | - |
|  | CVM N29710 | CP004066.1 | 1.73 | 31.4 | 23959310 |
|  | RM5611 | CP007179.1 | 1.73 | 31.3 | - |
|  | 15-537360 | CP006702.1 | 1.69 | 31.4 | 24336384 |
| *C. concisus* | 13826 | CP000792.1 | 2.1 | 39.2 | - |
| *C. corcagiensis* | CIT045 | JFAP00000000.1 | 1.67 | 31.9 | 24744327 |
| *C. cuniculorum* | DSM 23162 | JHZL00000000.1 | 1.87 | 31.3 | - |
| *C. curvus* | 525.92 | CP000767.1 | 1.97 | 44.5 | - |
| *C. fetus* subsp. *fetus* | 04/554 | CP008808.1 | 1.83 | 33.1 | 25232170 |
|  | 82-40 | CP000487.1 | 1.77 | 33.3 | - |
| *C. fetus* subsp. *testudinum* | 03-427 | CP006833.1 | 1.78 | 33.1 | 24336365 |
|  | pet-3 | CP009226.1 | 1.78 | 33.1 | 25700400 |
| *C. fetus* subsp. *venerealis* | 97/608 | CP008810.1 | 2 | 33.2 | 25232170 |
|  | cfvi03/293 | CP006999.1 | 2 | 33.1 | 24503995 |
|  | 84-112 | HG004426.1 | 1.99 | 33.2 | 24416416 |
| *C. gracilis* | ATCC 33236 | CP012196.1 | 2.28 | 46.6 | 26383656 |
| *C. hepaticus* | HV10 | NZ_LUKK00000000.1 | 1.48 | 27.9 | - |
| *C. hominis* | ATCC BAA-381 | CP000776.1 | 1.71 | 31.7 | - |
| *C. hyointestinalis* subsp. *hyointestinalis* | DSM 19053 | JHQP00000000.1 | 1.73 | 33.9 | - |
|  | LMG 9260 | CP015575.1 | 1.75 | 34 | 27417840 |
| *C. hyointestinalis* subsp. *lawsonii* | LMG 15993 | CP015576.1 | 1.75 | 33.5 | 27417840 |
| *C. iguaniorum* | 1485E | CP009043.1 | 1.75 | 35.9 | 25146144 |
| *C. insulaenigrae* | NCTC 12927 | CP007770.1 | 1.47 | 28.2 | 25381664 |
| *C. jejuni* subsp. *doylei* | 269.97 | CP000768.1 | 1.85 | 30.6 | - |
| *C. jejuni* subsp. *jejuni* | NCTC 11168 | AL111168.1 | 1.64 | 30.5 | 10688204; 17565669 |
|  | 81116 | CP000814.1 | 1.63 | 30.5 | 17873037 |
|  | 81-176 | CP000538.1 | 1.7 | 30.5 | - |
|  | 00-1597 | CP010306.1 | 1.74 | 30.4 | - |
|  | IA3902 | CP001876.1 | 1.67 | 30.5 | 22188995; 24201373 |
|  | F38011 | CP006851.1 | 1.69 | 30.5 | - |
|  | RM1221 | CP000025.1 | 1.78 | 30.3 | 15660156 |
|  | S3 | CP001960.1 | 1.72 | 30.5 | 21217004 |
|  | M1 | CP001900.1 | 1.62 | 30.6 | 20865039 |
|  | 32488 | CP006006.1 | 1.7 | 30.5 | - |
|  | 4031 | HG428754.1 | 1.67 | 30.5 | 25196593 |
|  | MTVDSCj20 | CP008787.1 | 1.65 | 30.5 | 25146148 |
|  | YH001 | CP010058.1 | 1.71 | 30.5 | 25657275 |
|  | 00-6200 | CP010307.1 | 1.67 | 30.5 | - |
|  | 01-1512 | CP010072.1 | 1.83 | 30.4 | - |
|  | 00-0949 | CP010301.1 | 1.83 | 30.4 | - |
|  | 35925B2 | CP010906.1 | 1.61 | 30.7 | - |
|  | 00-2425 | CP006729.2 | 1.72 | 30.5 | 27912729 |
|  | 00-2426 | CP006708.2 | 1.68 | 30.5 | 27912729 |
|  | 00-2538 | CP006707.2 | 1.72 | 30.5 | 27912729 |
|  | 00-2544 | CP006709.2 | 1.77 | 30.5 | 27912729 |
|  | CG8421 | CP005388.1 | 1.64 | 30.5 | 18809665 |
|  | ICDCCJ07001 | CP002029.1 | 1.71 | 30.6 | 21124772; 20455754 |
|  | PT14 | CP003871.3 | 1.64 | 30.5 | 24265498; 25601859 |
|  | R14 | CP005081.1 | 1.8 | 30.4 | - |
| *C. lari* | Slaughter Beach | CP011372.1 | 1.56 | 29.8 | 26472835 |
|  | CCUG 22395 | CP007776.1 | 1.52 | 29.9 | 25381664 |
|  | NCTC 11845 | CP007775.1 | 1.79 | 29.4 | 25381664 |
|  | RM16701 | CP007777.1 | 1.52 | 29.9 | 25381664 |
|  | RM16712 | CP007778.1 | 1.57 | 29.7 | 25381664 |
|  | RM2100 | CP000932.1 | 1.57 | 29.6 | 18713059; 25381664 |
| *C. lari* subsp. *concheus* | LMG 11760 | CP007771.1 | 1.5 | 29.7 | 25381664 |
| *C. mucosalis* | DSM 21682 | JHQQ00000000.1 | 1.75 | 36.6 | - |
| *C. ornithocola* | WBE38 | NZ_LXSU00000000.1 | 1.67 | 29.6 | 28126040 |
| *C. peloridis* | LMG 23910 | CP007766.1 | 1.76 | 28.4 | 25381664 |
| *C. pinnipediorum* | RM17260 | CP012546.1 | 1.78 | 30.4 | - |
|  | RM17261 | CP012547.1 | 1.74 | 30.4 | - |
|  | RM17262 | CP012548.1 | 1.64 | 30.3 | - |
|  | M203/00/3 | NZ_MBGA00000000.1 | 1.67 | 30.2 | - |
| *C. rectus* | RM3267 | ACFU00000000.1 | 2.51 | 44.8 | - |
| *C. showae* | RM3277 | ACVQ00000000.1 | 2.07 | 45.7 | - |
|  | CC57C | AOTD00000000.1 | 2.19 | 45.4 | 23409253 |
|  | UNSWCD | AMZQ00000000.1 | 2.13 | 45.1 | 23409253 |
| *C. sputorum* subsp. *sputorum* | INTA08/209 | JMTI00000000.1 | 1.78 | 29.1 | 25193310 |
| *C. subantarcticus* | LMG 24377 | CP007773.1 | 1.85 | 29.8 | 25381664 |
|  | LMG 24374 | CP007772.1 | 1.78 | 29.9 | 25381664 |
| *C. upsaliensis* | DSM 5365 | JHZN00000000.1 | 1.62 | 35 | - |
|  | JV21 | AEPU00000000.1 | 1.65 | 35 | - |
|  | RM3195 | AAFJ00000000.1 | 1.77 | 34.3 | 15660156 |
| *C. ureolyticus* | RIGS 9880 | CP012195 | 1.64 | 29.2 | 26543122 |
| *C. volucris* | LMG 24379 | CP007774.1 | 1.52 | 28.6 | 25381664 |
| *C. hepaticus* | S10-0209 | ERS1508458 | 1.54 | 28.5 | Crawshaw et al., 2015 |
|  | S11-010 | ERS1508462 | 1.59 | 28.5 | Crawshaw et al., 2015 |
|  | S11-5013 | ERS1508464 | 1.55 | 28.5 | Crawshaw et al., 2015 |
|  | S12-1018 | ERS1508467 | 1.53 | 28.2 | Crawshaw et al., 2015 |
|  | S11-0036 | ERS1508459 | 1.49 | 28.4 | This study |
|  | S11-0038 | ERS1508463 | 1.50 | 28.4 | Crawshaw et al., 2015 |
|  | S12-002 | ERS1508465 | - | - | Crawshaw et al., 2015 |
|  | S11-0069 | ERS1508460 | 1.49 | 29.1 | Crawshaw et al., 2015 |
|  | S11-0071 | ERS1508461 | 1.51 | 28.5 | Crawshaw et al., 2015 |
|  | S12-0322 | ERS1508466 | 1.53 | 28.4 | Crawshaw et al., 015 |
|  | HV10 | NZ_LUKK01000001.1 | 1.48 | 27.9 | [Van et al., 2016](#_ENREF_47) |
