# Supplementary material for: Genome Reduction for Niche Association in Campylobacter Hepaticus, A Cause of Spotty Liver Disease in Poultry
Source: Front Cell Infect Microbiol. 2017 Aug 11;7:354. doi: 10.3389/fcimb.2017.00354 (PMC5554493; doi:10.3389/fcimb.2017.00354)
Supplement: Supplementary Table S2 — Comparison of the Carbohydrates subsystem in C. hepaticus and C. jejuni (reference) genomes using RAST. [file Table2.DOC]

**Supplement Table S2.** **Comparison of the Carbohydrates subsystem in *C. hepaticus* and *C. jejuni* (reference) genomes using RAST.** *C. hepaticus* isolates: draft Australian *C. hepaticus* genome HV10. UK isolates: S10-0209, S11-010, S11-5013, S12-1018, S11-0036, S11-0038, S12-002, S11-0071 and S12-0322 Reference genomes: *C. jejuni* NCTC 11168, M1, PT14, R14 and 4031. The 4 different carbohydrate subsystems are indicated in bold. The subsequent pathways belonging to each of the subsystem are indicated with asterix (*).

| RAST subsystems | S10-209 | S11-010 | S11-5013 | S12-1018 | S11-036 | S11-038 | S11-069 | S11-071 | S12-0322 | HV-10 | Cj-M1 | Cj-11168 | Cj-PT14 | Cj-R14 | Cj-4031 |
| --- | --- | --- | --- | --- | --- | --- | --- | --- | --- | --- | --- | --- | --- | --- | --- |
| Farm | 1 | 1 | 1 | 1 | 2 | 2 | 4 | 4 | 5 |  |  |  |  |  |  |
| **Carbohydrates** | 112 | 112 | 112 | 112 | 84 | 84 | 86 | 86 | 82 | 83 | 62 | 62 | 62 | 75 | 68 |
| **Central carbohydrate metabolism** | 61 | 61 | 61 | 61 | 61 | 61 | 63 | 63 | 58 | 60 | 42 | 43 | 43 | 43 | 44 |
| Pyruvate metabolism II: acetyl-CoA, acetogenesis from pyruvate* | 11 | 11 | 11 | 11 | 11 | 11 | 11 | 11 | 9 | 11 | 4 | 1 | 4 | 4 | 4 |
| Entner-Doudoroff Pathway * | 10 | 10 | 10 | 10 | 10 | 10 | 10 | 10 | 10 | 10 | 0 | 0 | 0 | 0 | 0 |
| **One-carbon Metabolism** | 27 | 27 | 27 | 27 | 5 | 5 | 5 | 5 | 5 | 5 | 4 | 4 | 4 | 4 | 4 |
| Serine-glyoxylate cycle * | 22 | 22 | 22 | 22 | 0 | 0 | 0 | 0 | 0 | 0 | 0 | 0 | 0 | 0 | 0 |
| **Sugar alcohols** | 10 | 10 | 10 | 10 | 10 | 10 | 10 | 10 | 11 | 10 | 0 | 0 | 0 | 0 | 0 |
| Glycerol and Glycerol-3-phosphate Uptake and Utilization * | 10 | 10 | 10 | 10 | 10 | 10 | 10 | 10 | 11 | 10 | 0 | 0 | 0 | 0 | 0 |
| **Fermentation** | 2 | 2 | 2 | 2 | 2 | 2 | 2 | 2 | 2 | 2 | 5 | 5 | 5 | 5 | 5 |
| Fermentations: Lactate * | 0 | 0 | 0 | 0 | 0 | 0 | 0 | 0 | 0 | 0 | 3 | 3 | 3 | 3 | 3 |
